# The Differential Effects of Age on the Association Between Childhood Socioeconomic Disadvantage and Subjective Symptoms of Dementia Among Older Japanese People

**DOI:** 10.2188/jea.JE20180002

**Published:** 2019-07-05

**Authors:** Hiroshi Murayama, Mika Sugiyama, Hiroki Inagaki, Chiaki Ura, Fumiko Miyamae, Ayako Edahiro, Keiko Motokawa, Tsuyoshi Okamura, Shuichi Awata

**Affiliations:** 1 Institute of Gerontology, The University of Tokyo, Tokyo, Japan; 2 Research Team for Promoting Independence and Mental Health, Tokyo Metropolitan Institute of Gerontology, Tokyo, Japan

**Keywords:** dementia, subjective symptoms, childhood, socioeconomic status, lifecourse, Japan

## Abstract

**Background:**

Despite increasing evidence of an association between childhood socioeconomic disadvantage and cognitive outcomes, such as dementia and cognitive decline, in Western countries, there are no studies on this association from non-Western societies. We investigated the relationship between childhood socioeconomic status (SES) and subjective symptoms of dementia among community-dwelling older Japanese people and examined age and sex variations in this association.

**Methods:**

Data were derived from a cross-sectional survey for all community-dwelling individuals aged 65 years and over in Adachi, Tokyo (*n* = 132,005). We assessed subjective dementia symptoms using a self-administered dementia checklist, which was validated by comparison with the Clinical Dementia Rating scale.

**Results:**

Data from 75,358 questionnaires were analyzed. After adjusting for potential covariates, lower childhood SES was associated with greater likelihood of subjective dementia symptoms. We found a significant interaction between childhood SES and age on subjective dementia symptoms but no interaction between childhood SES and sex. Age-stratified analysis indicated that the association between lower childhood SES and subjective dementia symptoms was stronger in the ≥75 years subgroup than in the 65–74 years subgroup, indicating an effect modification of age on this association.

**Conclusions:**

Our findings suggested that low SES in childhood might have a long-term influence on dementia symptoms in late life and that this influence varied by age. This differential association might be explained by the social and historical context in Japan (ie, World War II, postwar chaos, and high economic growth) that has shaped participants’ early experiences.

## INTRODUCTION

The estimated total worldwide cost of dementia was 818 billion United States dollars in 2015 and this is expected to rise to 2 trillion dollars by 2030.^1^ The development of a dementia prevention strategy is a current global public health issue. Dementia onset is a long-term process and is affected by various factors, such as age, low education, smoking, obesity, physical inactivity, comorbidities (eg, hypertension, diabetes mellitus, and depression), and drug use.^2^^–^^5^ However, most research has focused on these processes during mid- and late-life.

Increasing evidence indicates the usefulness of a lifecourse perspective on the process of cognitive decline and dementia onset, focusing on childhood socioeconomic disadvantage.^6^^–^^13^ For example, Moceri et al showed that children from larger households and whose fathers were unskilled manual workers had a greater risk of Alzheimer’s disease.^6^^,^^7^ Moreover, Turrell et al reported that childhood lower socioeconomic conditions, lower education, and lower income were associated with poorer cognitive performance.^10^

These studies suggest a persistent influence of childhood socioeconomic status (SES) on cognitive function and dementia onset in old age. However, most previous studies on the relationship between childhood SES and cognitive outcomes have been conducted in Western countries. Considering the differences in social, cultural, and historical contexts between Western and non-Western countries, it is important to explore the association between childhood SES and cognitive outcomes in non-Western societies, such as Japan. Many older people in Japan experienced potentially harmful circumstances resulting from war, such as food shortages, poverty, military enlistment, injury, and the death of family members in the periods during World War II (WWII) and immediately after WWII (a chaotic postwar era).^14^^–^^16^ These social and historical features may influence the association between childhood SES and health in Japanese individuals.^17^^–^^20^ Data from non-Western societies include factors not present in Western countries and could offer useful insights into the underlying mechanisms and prevention strategies of dementia.

In the current study, we aimed to investigate the association between childhood SES and subjective symptoms of dementia among community-dwelling older Japanese people using a population-based, large-scale questionnaire survey. Some Japanese studies have shown effect modifications of age and sex on the association between childhood socioeconomic disadvantage and late-life health status factors, such as mortality,^18^ depressive mood,^19^ and functional impairment.^17^^,^^20^ Therefore, we also examined age and sex variations in the association between childhood SES and subjective dementia symptoms.

## METHODS

### Study design, participants, and setting

A cross-sectional survey was conducted in July 2015 using a mailed self-administered questionnaire. The target population comprised all community-dwelling individuals aged 65 years and over in Adachi Ward, Tokyo, Japan. Adachi is located in the northeast part of the Tokyo metropolitan area. As of July 1, 2015, its population was 677,531 (339,951 men and 337,580 women), and it had a population density of 12,723.6 persons/km^2^. The proportion of people aged 65 years and over was 24.3%. Adachi has the fifth biggest population size but the fifth lowest population density among the 23 wards in the Tokyo metropolitan area. The average taxable income of Adachi is the lowest among the 23 wards.

As of April 1, 2015, the population of individuals aged 65 years and over was 163,719. We excluded people with long-term care insurance certification, including both required support levels 1–2 and care levels 1–5 (ie, people who the local municipality had officially certified as requiring any type of long-term care services^21^; *n* = 29,327) and those who had died, moved away, or had newly received long-term care insurance certification by the date the questionnaire was dispatched in July (*n* = 2,387). Therefore, 132,005 questionnaires were distributed. To combine the data from the questionnaire survey with data from other sources (eg, basic resident register, death data, and long-term care insurance certification), we added a label with an identification number and the participant’s name and home address to the questionnaire. However, all analyses preserved the anonymity of the data.

Most of the study participants were children during WWII (1937–1945) and during the chaotic postwar era. The year that WWII ended (1945) was the birth year of people aged 70 years in 2015. Those aged 65–74 years in 2015 were of school age (around 7–12 years old) immediately before and during a period of high economic growth in Japan (mid-1950s to mid-1970s). In contrast, those aged 75 years and over were of school age during the prewar, WWII, and postwar periods.

The study protocol was approved by the Ethics Committee of Tokyo Metropolitan Institute of Gerontology. All participants gave informed consent before their inclusion in the study.

### Measures

#### Subjective symptoms of dementia

Subjective symptoms of dementia were assessed using a self-administered dementia checklist (SDC), which was developed to enable community-dwelling older people to recognize their declining functions at an early stage of dementia progression.^22^^,^^23^ This scale consists of 10 items on two subscales (subjective cognitive decline and instrumental activities of daily living); both subjective cognitive decline and instrumental activities of daily living can predict onset of dementia.^24^^,^^25^ Item responses are on a four-point Likert scale. The total score range is 10–40; higher scores indicate greater severity of subjective dementia symptoms.

The SDC discriminates between patients with dementia (Clinical Dementia Rating [CDR] scale score of ≥1) and those without dementia (CDR score of 0 or 0.5) using a cut-off point of 17/18.^22^^,^^23^ The CDR is a global rating scale that evaluates the severity of symptoms of dementia. During the development of the SDC, psychiatrists interviewed older individuals and members of their family and used their clinical judgment to assign CDR scores. We regarded SDC scores of ≥18 as indicating subjective dementia symptoms and used this cut-off point in the study.

This study was a population-based, large-scale survey of community-dwelling older people; therefore, the use of the CDR was infeasible. Instead, as the SDC had been validated via comparison with the CDR (as scored by psychiatrists), we used the SDC to assess subjective dementia symptoms as a surrogate marker of dementia.

#### Childhood socioeconomic disadvantage

We asked participants to retrospectively rate their childhood SES using a single item that asked: “How would you rate the socioeconomic status of your family when you were at school age, in comparison with other families?”. Responses were indicated on a five-point Likert scale: “high,” “middle-high,” “middle,” “middle-low,” and “low.” Because the proportion of responses in the “high” category was small (2.6%), we combined the “high” and “middle-high” categories. In total, we examined four categories in our final analysis.

#### Covariates

We included age, sex, years of education, marital status, living alone, current working status, smoking status, regular exercise habits, body mass index, presence of diagnosed diseases, and depression. The local municipality (ie, Adachi Ward) provided us with information about age and sex from the basic resident register. The questionnaire assessed the other variables. Years of education were categorized into three groups (≤9 years, 10–12 years, and ≥13 years). Body mass index (kg/m^2^) was divided into three categories (underweight [<18.5 kg/m^2^], normal weight [18.5–24.9 kg/m^2^], and overweight [≥25 kg/m^2^]). Diagnosed diseases were cancer, heart disease, stroke, hypertension, hyperlipidemia, and diabetes mellitus. To assess depression, we used a two-question case-finding instrument for depression.^26^ The validity of this instrument to detect depression has been demonstrated.

### Statistical analysis

Logistic regression analysis was used to examine the association between childhood SES and the likelihood of subjective dementia symptoms. We used a four-step modeling strategy. In model 1, we included age, sex, and childhood SES. In model 2, years of education, marital status, living alone, and working status were added to model 1. In model 3, smoking status, regular exercise habits, and body mass index were additionally controlled. Finally, presence of diagnosed diseases and depression were adjusted in model 4. Moreover, to determine whether there were age and sex variations in the association between childhood SES and subjective dementia symptoms, model 4 additionally explored the interaction between childhood SES and age/sex. When significant interactions were found, we additionally performed stratified analysis by age and/or sex. The results of the estimations were shown as odds ratios (ORs) with 95% confidence intervals (CIs). All analyses were performed using IBM SPSS 23 (IBM Corp., Armonk, NY, USA).

## RESULTS

Of 132,005 questionnaires distributed, 78,917 were returned (response rate: 59.8%). We compared respondents and non-respondents on age and sex. A lower proportion of respondents were men, and respondents were older than non-respondents (45.0% vs 49.0%, and mean 73.8 [standard deviation {SD}, 6.0] years vs 72.4 [SD, 6.0] years; *P* < 0.001 using chi-square test and *t*-test, respectively). Because this study targeted community-dwelling older people, we excluded participants who were not living in their own homes (eg, those currently hospitalized for a long time or who resided in care facilities; *n* = 3,559). As a result, 75,358 questionnaires were regarded as valid responses (valid response rate: 57.1%) and were included in the analysis.

Table 1 shows participants’ characteristics. The average age was 73.8 (SD, 6.0) years, and 45.0% of participants were men. In terms of childhood SES, the proportion of responses for the high/middle-high, middle, middle-low, and low categories were 15.1%, 45.9%, 26.0%, and 13.1%, respectively. As assessed using the SDC, 6.6% of participants were categorized as having subjective dementia symptoms, and the proportion was greater in the older subgroup.

**Table 1.** Participant characteristics

	Total	65–74 years	≥75 years
	*n* = 75,358	*n* = 43,790	*n* = 31,568
Age, years	73.8 (6.0)	69.5 (3.0)	79.6 (3.8)
Men	45.0	45.3	44.5
Years of education			
≤9	37.4	30.6	47.3
10–12	39.9	43.1	35.2
≥13	22.7	26.2	17.4
Married	65.8	69.7	60.2
Living alone	22.0	20.6	23.8
Currently working	37.7	46.8	24.8
Current smoker	13.2	16.6	8.5
Regularly exercising	57.4	58.4	56.0
Body mass index, kg/m^2^			
<18.5	7.3	6.6	8.4
18.5–24.9	68.7	68.8	68.5
≥25.0	23.9	24.6	23.1
Diagnosed disease			
Cancer	9.3	8.6	10.3
Heart disease	11.1	8.9	13.8
Stroke	4.1	3.6	4.8
Hypertension	45.8	42.6	50.4
Hyperlipidemia	12.2	13.0	11.0
Diabetes mellitus	14.5	14.3	14.9
Depression	19.5	16.8	23.2
Childhood SES			
High/middle-high	15.1	14.0	16.5
Middle	45.9	44.5	47.8
Middle-low	26.0	27.5	23.8
Low	13.1	14.0	11.9
Subjective dementia symptoms	6.6	3.7	10.6

SES, socioeconomic status.

Values represent mean (standard deviation) or %.

Table 2 indicates the association between childhood disadvantage and subjective dementia symptoms. In model 1, after adjusting for age and sex only, lower childhood SES was associated with greater likelihood of having subjective dementia symptoms (OR 2.21; 95% CI, 1.97–2.48 for the low category, OR 1.69; 95% CI, 1.52–1.88 for the middle-low category, and OR 1.13; 95% CI, 1.02–1.25 for the middle category). This association was attenuated in models 2 through 4, but low and middle-low childhood SES were still statistically significant even in the final model (OR 1.38; 95% CI, 1.19–1.60 for the low category and OR 1.22; 95% CI, 1.07–1.39 for the middle-low category).

**Table 2.** Associations between childhood socioeconomic status and subjective dementia symptoms

	Model 1		Model 2		Model 3		Model 4	
			
	OR	(95% CI)	OR	(95% CI)	OR	(95% CI)	OR	(95% CI)
Age, every 10 years	3.25	(3.10–3.42)	2.78	(2.62–2.95)	2.58	(2.43–2.75)	2.52	(2.36–2.69)
Men	1.33	(1.24–1.41)	1.48	(1.38–1.60)	1.55	(1.43–1.67)	1.53	(1.41–1.67)
Childhood SES								
Low	2.21	(1.97–2.48)	1.77	(1.54–2.02)	1.70	(1.48–1.95)	1.38	(1.19–1.60)
Middle-low	1.69	(1.52–1.88)	1.41	(1.25–1.59)	1.36	(1.20–1.55)	1.22	(1.07–1.39)
Middle	1.13	(1.02–1.25)	0.99	(0.89–1.11)	0.99	(0.88–1.11)	0.98	(0.87–1.11)
High/middle-high	1.00		1.00		1.00		1.00	
Years of education								
≤9			1.54	(1.39–1.71)	1.48	(1.33–1.65)	1.44	(1.28–1.61)
10–12			1.13	(1.02–1.26)	1.09	(0.98–1.22)	1.08	(0.96–1.21)
≥13			1.00		1.00		1.00	
Not married			1.20	(1.09–1.32)	1.13	(1.02–1.28)	1.16	(1.04–1.29)
Living alone			0.66	(0.59–0.74)	0.69	(0.61–0.78)	0.61	(0.53–0.69)
Currently not working			1.75	(1.61–1.91)	1.82	(1.67–1.99)	1.60	(1.46–1.75)
Current smoker					0.99	(0.89–1.11)	0.93	(0.82–1.04)
No regular exercise					2.59	(2.40–2.79)	2.20	(2.04–2.39)
Body mass index, kg/m^2^								
<18.5					1.46	(1.29–1.65)	1.33	(1.17–1.51)
18.5–24.9					1.00		1.00	
≥25.0					1.02	(0.93–1.11)	1.02	(0.93–1.12)
Cancer							1.03	(0.92–1.16)
Heart disease							1.25	(1.13–1.39)
Stroke							1.91	(1.66–2.19)
Hypertension							0.93	(0.86–1.01)
Hyperlipidemia							1.02	(0.91–1.14)
Diabetes mellitus							1.40	(1.27–1.54)
Depression							5.48	(5.07–5.91)

CI, confidence interval; OR, odds ratio; SES, socioeconomic status.

We examined the differential effect of age/sex on the association between childhood SES and subjective dementia symptoms by adding an interaction between childhood SES and age/sex on subjective dementia symptoms to model 4 (data not shown in the table). Statistically significant interaction terms between childhood SES and age were observed (*P* = 0.030 and *P* = 0.015 for low × age and middle-low × age, respectively; *P* = 0.193 for middle × age). This indicated an effect modification of age on the association between childhood SES and subjective dementia symptoms. However, there was no significant interaction between childhood SES and sex (*P* = 0.131, *P* = 0.269, and *P* = 0.322 for low × sex, middle-low × sex, and middle × sex, respectively).

To examine the effect modification of age, we conducted a stratified analysis by age subgroup (65–74 years and ≥75 years); the results are shown in Table 3. The association between childhood SES and subjective dementia symptoms was statistically significant in both age subgroups in model 4. However, this association was greater in the ≥75 years subgroup than in the 65–74 years subgroup.

**Table 3.** Age-stratified analysis on the associations between childhood socioeconomic status and subjective dementia symptoms

	Model 1		Model 2		Model 3		Model 4	
			
	OR	(95% CI)	OR	(95% CI)	OR	(95% CI)	OR	(95% CI)
**65–74 years**								
Childhood SES								
Low	2.06	(1.69–2.51)	1.66	(1.33–2.08)	1.60	(1.27–2.02)	1.32	(1.04–1.68)
Middle-low	1.51	(1.25–1.82)	1.31	(1.06–1.61)	1.30	(1.05–1.61)	1.21	(0.97–1.51)
Middle	0.95	(0.79–1.15)	0.87	(0.71–1.07)	0.88	(0.72–1.09)	0.93	(0.75–1.15)
High/middle-high	1.00		1.00		1.00		1.00	
**≥75 years**								
Childhood SES								
Low	2.30	(1.99–2.67)	1.85	(1.56–2.19)	1.78	(1.49–2.12)	1.46	(1.21–1.76)
Middle-low	1.81	(1.59–2.07)	1.49	(1.29–1.73)	1.42	(1.22–1.66)	1.24	(1.06–1.46)
Middle	1.23	(1.09–1.39)	1.07	(0.93–1.23)	1.06	(0.92–1.22)	1.03	(0.89–1.20)
High/middle-high	1.00		1.00		1.00		1.00	

CI, confidence interval; OR, odds ratio; SES, socioeconomic status.

Model 1: Adjusted for age, sex, and childhood SES.

Model 2: Addition of years of education, marital status, living alone, and current working status to model 1.

Model 3: Addition of smoking status, regular exercise, and body mass index to model 2.

Model 4: Addition of cancer, heart disease, stroke, hypertension, hyperlipidemia, diabetes mellitus, and depression to model 3.

## DISCUSSION

This study examined the association between childhood socioeconomic disadvantage and the likelihood of subjective dementia symptoms (as assessed using the SDC) among community-dwelling older Japanese people. Despite increasing evidence for an association between childhood socioeconomic disadvantage and cognitive outcomes in Western societies,^6^^–^^13^ there are no studies from Asian countries. Therefore, it is important to obtain evidence from Asian samples to inform a lifecourse approach to dementia prevention. To our knowledge, this is the first report of an association between childhood SES and dementia-related outcome in Asian countries.

We found that lower childhood SES was associated with greater likelihood of having subjective dementia symptoms in later life, even after controlling for potential covariates in models 1–4. This direct association could be explained via biological embedding, the process by which the effects of experience become deeply embedded and alter human biology and development.^27^ Biological embedding can also influence brain reserve. For example, childhood socioeconomic disadvantage can hamper the development of crucial brain structures. If the brain does not fully develop during maturation, there may be an increased risk of Alzheimer’s disease later in life.^6^ Indeed, lower childhood SES is associated with smaller adult hippocampal volume.^28^ This indicates that early life conditions might affect structural brain development and then cause dementia symptoms in later life.

Because we found a significant interaction between childhood SES and age on subjective dementia symptoms, we performed an age-stratified analysis. This showed that the association between childhood SES and subjective dementia symptoms was stronger in old-old participants than in young-old participants in models 1 to 4. Although age was found to be strongly associated with subjective dementia symptoms in this study and in previous studies,^2^^–^^5^ we revealed that age is also an effect modifier of the association between childhood SES and dementia symptoms. Participants aged ≥75 years in 2015 experienced severe childhood conditions (eg, food shortages, poverty, military enlistment, injury, and the death of family members) during wartime or immediately after WWII (a chaotic postwar era). Biological embedding might be common in this generation, causing a direct association between lower childhood SES and subjective dementia symptoms in late life. In contrast, participants aged 65–74 years were of school age just before or at the start of the high economic growth period. In this period, the living standards of Japanese people increased and socioeconomic inequality decreased.^29^ Thus, the direct influence of childhood SES on dementia symptoms might be relatively smaller in the 65–74 years subgroup than in the ≥75 years subgroup. Systematic differences in early experience across different socioeconomic environments could lead to different biological outcomes, which in turn may influence cognitive function across the life cycle.

The present study has several limitations. First, we assessed subjective dementia symptoms using the SDC. This scale is based on self-reported responses rather than on physician diagnosis. In addition, although the SDC can discriminate patients with dementia (CDR score of ≥1) from those without dementia,^22^^,^^23^ a previous study indicates that the discriminant validity of this scale is insufficient.^22^ Therefore, it would be useful to conduct further studies of individuals with diagnosed dementia to confirm the findings reported here. Second, we assessed childhood SES retrospectively. Although the validity of using retrospective assessments of childhood SES in studies of life course epidemiology has been confirmed,^30^ the effects of such assessments on outcomes might have been underestimated.^31^ Third, as our findings were derived from a questionnaire survey with an approximate response rate of only 60%, we cannot rule out the possibility of selection bias. For example, people with severe dementia symptoms, childhood disadvantage, and/or disability may be less likely to respond to surveys. Thus, the current results may have underestimated the associations between childhood SES and subjective dementia symptoms. Fourth, there may be residual confounding of the association between childhood SES and subjective dementia symptoms. For example, parental educational level and parental dementia history might affect both participants’ childhood SES and their probability of having dementia symptoms. These factors should be considered in future studies.

In conclusion, using a population-based, large-scale questionnaire survey, we found that lower SES in childhood was associated with greater likelihood of having subjective dementia symptoms in later life. The current findings highlight the importance of considering long-term effects of childhood socioeconomic environment in assessing the risks of adverse health outcomes. Furthermore, this association varied by age. This differential association between childhood SES and subjective dementia symptoms might be explained by the social and historical context in Japan (ie, WWII, postwar chaos, and high economic growth) that shaped participants’ early experiences. Studies from Western countries have also reported an association between childhood socioeconomic disadvantage and cognitive outcomes. However, the social and historical backgrounds that may give rise to this association differ across societies and nations. Thus, policy-makers should develop upstream interventions that target childhood, such as enhancing social security in early age, depending on the background of the particular society. Because childhood SES may directly affect dementia symptoms in later life, such interventions could contribute to reducing the incidence of future dementia.

## References

[1] Wimo A, Guerchet M, Ali GC, The worldwide costs of dementia 2015 and comparisons with 2010. Alzheimers Dement. 2017;13:1–7. 10.1016/j.jalz.2016.07.150 27583652 PMC5232417

[2] Chen JH, Lin KP, Chen YC Risk factors for dementia. J Formos Med Assoc. 2009;108:754–764. 10.1016/S0929-6646(09)60402-2 19864195

[3] Plassman BL, Williams JW Jr, Burke JR, Holsinger T, Benjamin S Systematic review: factors associated with risk for and possible prevention of cognitive decline in later life. Ann Intern Med. 2010;153:182–193. 10.7326/0003-4819-153-3-201008030-00258 20547887

[4] Daviglus ML, Plassman BL, Pirzada A, Risk factors and preventive interventions for Alzheimer disease: state of the science. Arch Neurol. 2011;68:1185–1190. 10.1001/archneurol.2011.100 21555601

[5] Xu W, Tan L, Wang HF, Meta-analysis of modifiable risk factors for Alzheimer’s disease. J Neurol Neurosurg Psychiatry. 2015;86:1299–1306. 10.1136/jnnp-2015-310548 26294005

[6] Moceri VM, Kukull WA, Emanuel I, van Belle G, Larson EB Early-life risk factors and the development of Alzheimer’s disease. Neurology. 2000;54:415–420. 10.1212/WNL.54.2.415 10668705

[7] Moceri VM, Kukull WA, Emanual I, Using census data and birth certificates to reconstruct the early-life socioeconomic environment and the relation to the development of Alzheimers disease. Epidemiology. 2001;12:383–389. 10.1097/00001648-200107000-00007 11416775

[8] Kaplan GA, Turrell G, Lynch JW, Everson SA, Helkala EL, Salonen JT Childhood socioeconomic position and cognitive function in adulthood. Int J Epidemiol. 2001;30:256–263. 10.1093/ije/30.2.256 11369724

[9] Borenstein AR, Copenhaver CI, Mortimer JA Early-life risk factors for Alzheimer disease. Alzheimer Dis Assoc Disord. 2006;20:63–72. 10.1097/01.wad.0000201854.62116.d7 16493239

[10] Turrell G, Lynch JW, Leite C, Raghunathan T, Kaplan GA Socioeconomic disadvantage in childhood and across the life course and all-cause mortality and physical function in adulthood: evidence from the Alameda County Study. J Epidemiol Community Health. 2007;61:723–730. 10.1136/jech.2006.050609 17630374 PMC2653004

[11] Nguyen CT, Couture MC, Alvarado BE, Zunzunegui MV Life course socioeconomic disadvantage and cognitive function among the elderly population of seven capitals in Latin America and the Caribbean. J Aging Health. 2008;20:347–362. 10.1177/0898264308315430 18332188

[12] Fors S, Lennartsson C, Lundberg O Childhood living conditions, socioeconomic position in adulthood, and cognition in later life: exploring the associations. J Gerontol B Psychol Sci Soc Sci. 2009;64:750–757. 10.1093/geronb/gbp029 19420323

[13] Zeki Al Hazzouri A, Haan MN, Kalbfleisch JD, Galea S, Lisabeth LD, Aiello AE Life-course socioeconomic position and incidence of dementia and cognitive impairment without dementia in older Mexican Americans: results from the Sacramento area Latino study on aging. Am J Epidemiol. 2011;173:1148–1158. 10.1093/aje/kwq483 21430188 PMC3121319

[14] Oiso T Changing food patterns in Japan. Prog Clin Biol Res. 1981;77:527–538. 7335701

[15] Watanabe T Who became the soldiers? part 1: inequality in pre-war Showa period military service cohorts. Kwansei Gakuin Sociology Department Studies. 2014;119:1–18.

[16] Watanabe T Inequality in mortality risk during the Asia-Pacific War. Kwansei Gakuin Sociology Department Studies. 2016;123:85–101.

[17] Fujiwara T, Kondo K, Shirai K, Suzuki K, Kawachi I Associations of childhood socioeconomic status and adulthood height with functional limitations among Japanese older people: results from the JAGES 2010 Project. J Gerontol A Biol Sci Med Sci. 2014;69:852–859. 10.1093/gerona/glt189 24285745

[18] Tani Y, Kondo N, Nagamine Y, Childhood socioeconomic disadvantage is associated with lower mortality in older Japanese men: the JAGES cohort study. Int J Epidemiol. 2016;45:1226–1235. 10.1093/ije/dyw146 27401729 PMC5965916

[19] Tani Y, Fujiwara T, Kondo N, Childhood socioeconomic status and onset of depression among Japanese older adults: the JAGES prospective cohort study. Am J Geriatr Psychiatry. 2016;24:717–726. 10.1016/j.jagp.2016.06.001 27569265

[20] Murayama H, Fujiwara T, Tani Y, Long-term impact of childhood disadvantage on late-life functional decline among older Japanese: results from the JAGES prospective cohort study. J Gerontol A Biol Sci Med Sci. 2018;73(7):973–979. 10.1093/gerona/glx171 28957992

[21] Shirasawa M Current situation and issues of the long-term care insurance system in Japan. J Asian Health Policy. 2015;8:230–242. 10.1080/17516234.2015.1015208

[22] Miyamae F, Ura C, Sakuma N, The development of a self-administered dementia checklist: the examination of concurrent validity and discriminant validity. Nihon Ronen Igakkai Zasshi. 2016;53:354–362. 10.3143/geriatrics.53.354 27885222

[23] Ura C, Miyamae F, Sakuma N, Development of a self-administered dementia checklist: examination of factorial validity and internal reliability. Nihon Ronen Igakkai Zasshi. 2015;52:243–253. 10.3143/geriatrics.52.243 26268382

[24] Barberger-Gateau P, Commenges D, Gagnon M, Letenneur L, Sauvel C, Dartigues JF Instrumental activities of daily living as a screening tool for cognitive impairment and dementia in elderly community dwellers. J Am Geriatr Soc. 1992;40:1129–1134. 10.1111/j.1532-5415.1992.tb01802.x 1401698

[25] Kryscio RJ, Abner EL, Cooper GE, Self-reported memory complaints: implications from a longitudinal cohort with autopsies. Neurology. 2014;83:1359–1365. 10.1212/WNL.0000000000000856 25253756 PMC4189103

[26] Whooley MA, Avins AL, Miranda J, Browner WS Case-finding instruments for depression: two questions are as good as many. J Gen Intern Med. 1997;12:439–445. 10.1046/j.1525-1497.1997.00076.x 9229283 PMC1497134

[27] Hertzman C The biological embedding of early experience and its effects on health in adulthood. Ann N Y Acad Sci. 1999;896:85–95. 10.1111/j.1749-6632.1999.tb08107.x 10681890

[28] Staff RT, Murray AD, Ahearn TS, Mustafa N, Fox HC, Whalley LJ Childhood socioeconomic status and adult brain size: childhood socioeconomic status influences adult hippocampal size. Ann Neurol. 2012;71:653–660. 10.1002/ana.22631 22522480

[29] Minami R. *The economic development of Japan: a quantitative study*. London: Macmillan Press; 1986.

[30] Krieger N, Okamoto A, Selby JV Adult female twins’ recall of childhood social class and father’s education: a validation study for public health research. Am J Epidemiol. 1998;147:704–708. 10.1093/oxfordjournals.aje.a009512 9554610

[31] Batty GD, Lawlor DA, Macintyre S, Clark H, Leon DA Accuracy of adults’ recall of childhood social class: findings from the Aberdeen children of the 1950s study. J Epidemiol Community Health. 2005;59:898–903. 10.1136/jech.2004.030932 16166367 PMC1732932

